# Systemic BCG‐osis following intravesical BCG instillation for bladder carcinoma

**DOI:** 10.1002/ccr3.1129

**Published:** 2017-08-15

**Authors:** Frank Liaw, Yan Yu Tan, David Hendry

**Affiliations:** ^1^ Department of Urology Queen Elizabeth University Hospital Glasgow G51 4TF UK; ^2^ Department of Gastroenterology Monklands Hospital Airdrie ML6 0JS UK

**Keywords:** Adverse effects, Bacillus Calmette‐Guérin, BCG immunotherapy, case report, intravesical BCG, systemic BCG‐osis

## Abstract

Intravesical instillation of Bacillus Calmette‐Guérin (BCG) has been shown to be an effective form of immunotherapy for bladder cancer. This case report describes a patient who develops systemic BCG‐osis following intravesical BCG instillation and demonstrates the importance of being aware of more severe complications associated with BCG immunotherapy.

## Introduction

Eighty percent of bladder cancer, the second most common urological malignancy in the UK, can be classified as nonmuscle invasive bladder cancer (NMIBC). While adjuvant therapy may not be required for low‐grade papillary NMIBC tumors, studies have demonstrated how it is vital in management for higher grade tumors 1.

Intravesical instillation of Bacillus Calmette‐Guérin (BCG) has been shown to be an effective form of immunotherapy for bladder cancer 2, 3, 4. The role of adjuvant BCG immunotherapy in comparison with other intravesical agents such as mitomycin C, thiotepa, epirubicin, bleomycin, and cytosine arabinoside however remains unclear 5.

Originally developed as a live vaccine against tuberculosis, BCG has been demonstrated to significantly reduce the risk of progression following TURBT for superficial bladder cancer 6. While its exact mechanism of action within the bladder remains unconfirmed, it has been postulated that this is mainly due to a local immune response caused by exposure to BCG. Once bound to the bladder wall through an interaction between bacterial antigen 85 complex and fibronectin, BCG is taken up by urothelial, inflammatory, and tumor cells where it induces a T‐helper type 1 response detectable via the cytokine pattern within the urine 7. Alteration of the tumor cell phenotype via action of IFN‐gamma also causes tumor cells to become both lymphokine‐activated killer cell targets and antigen presenting cells 8.

## Case Report

We present the case of a 63‐year‐old man with G2 pT1c (high grade) papillary bladder transitional cell carcinoma who was admitted with a 3 week history of general malaise, fevers, night sweats, reduced appetite, and headaches.

He had completed the standard induction course of six weekly instillations of BCG as adjuvant therapy following TURBT and was given his first booster dose of BCG following which he became unwell.

On initial examination, he was noted to be clinically well. He was apyrexial and denied having any respiratory or urinary symptoms. However, laboratory results demonstrated acute kidney injury, mild transaminitis, pancytopaenia (neutrophils of 1.5 × 10^9^/L, platelets of 54 × 10^9^/L), and elevated C‐reactive protein of 111 mg/L (Table 1). Chest radiograph was unremarkable. He was started on intravenous fluids and cephalexin, which was later changed to intravenous amoxicillin and aztreonam for possible urological sepsis. The main differential diagnoses at this stage included urological sepsis and systemic BCG‐osis.

**Table 1:** Initial laboratory investigation results

Laboratory results	Value	Reference range
WBC (×10^9^/L)	2.5	4.0–11.0
RBC (×10^12^/L)	4.78	4.50–6.50
Hemoglobin (g/L)	138	130–180
MCV (fL)	81.8	80.0–100.0
Platelets (×10^9^/L)	54	150–400
Neutrophils (×10^9^/L)	1.5	2.0–7.5
Lymphocytes (×10^9^/L)	0.8	1.5–4.0
Monocytes (×10^9^/L)	0.1	0.2–0.8
Blood film	Hypergranular neutrophils. Genuine thrombocytopenia. Anisocytosis.	
Sodium (mmol/L)	132	133–146
Potassium (mmol/L)	3.4	3.5–5.3
Chloride (mmol/L)	103	95–108
Urea (mmol/L)	12.3	2.5–7.8
Creatinine (*μ*mol/L)	176	40–130
Estimated GFR (mL/min)	34	>60
Calcium (adjusted) (mmol/L)	2.45	2.20–2.60
Magnesium (mmol/L)	0.78	0.70–1.00
Phosphate (mmol/L)	1.47	0.80–1.50
Total Bilirubin (*μ*mol/L)	21	<20
ALT (U/L)	99	<50
AST (U/L)	85	<40
Alkaline phosphatase (U/L)	198	30–130
Albumin (g/L)	28	35–50
C‐reactive protein (mg/L)	111	0–10
ESR (mm/h)	8	1–10

**Figure 1:**
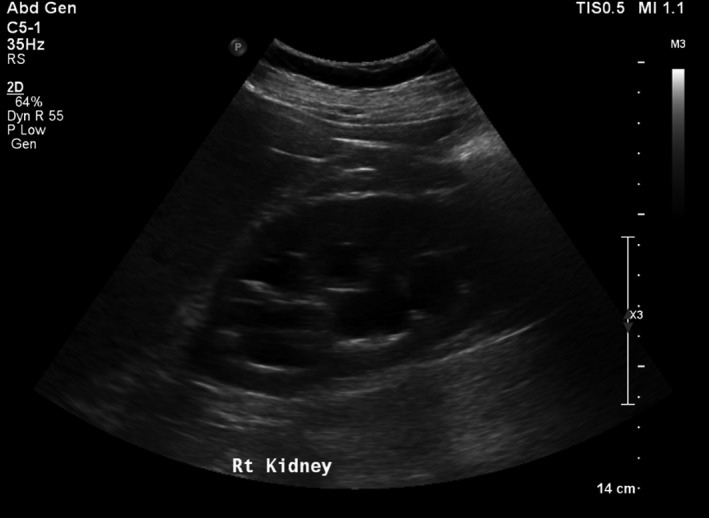
Right kidney hydronephrosis.

**Figure 2:**
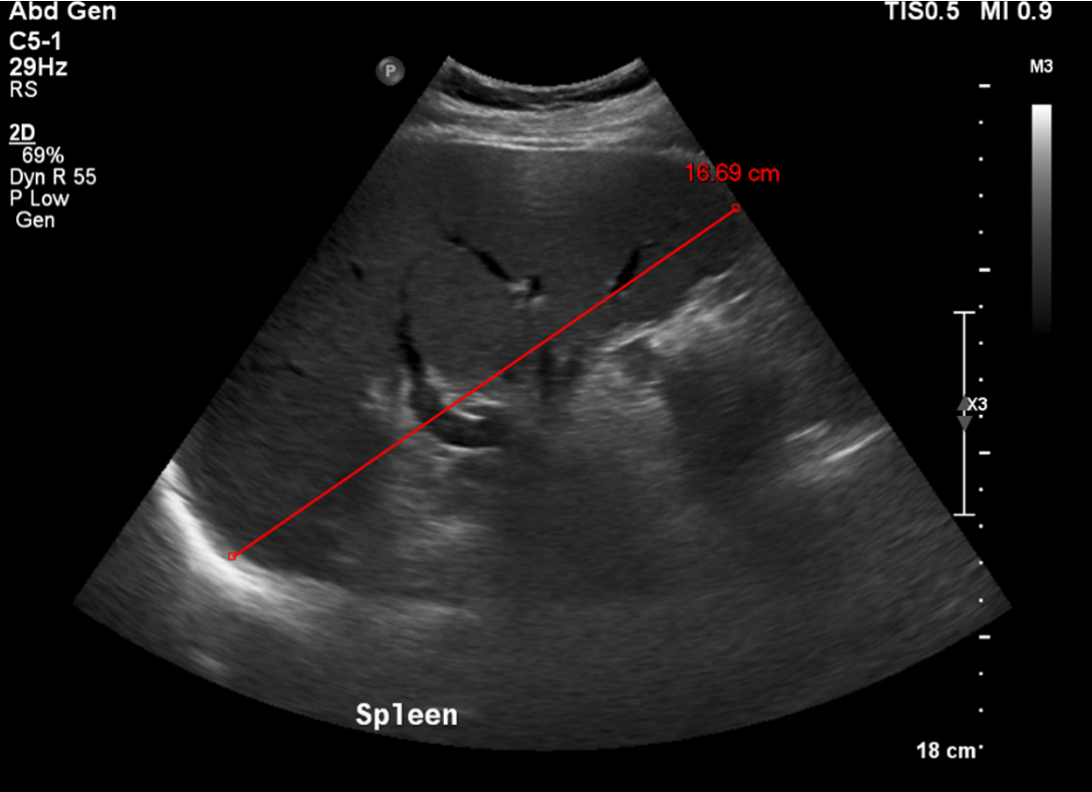
Mild splenomegaly.

Throughout his admission, he was noted to have swinging pyrexia up to a temperature of 39.8°C, but blood and urine cultures, viral gargle, along with sputum samples for AFB, returned negative. An abdominal ultrasound scan and computed tomography (CT) scan of abdomen and pelvis both demonstrated right kidney hydronephrosis (Fig. 1) and mild splenomegaly (Fig. 2).

As the patient failed to improve, he was commenced on intravenous piperacillin/tazobactam and gentamicin for neutropenic sepsis. At this point of his admission, there was growing suspicion that he had developed systemic BCG‐osis. The Infectious Diseases team suggested a cystoscopy with biopsies for pathology to ascertain whether granulomatous inflammation was present. However, it was felt there was a significant risk with cystoscopy and biopsy due to thrombocytopenia and neutropenia. In addition, given the fact that he had had a recent BCG instillation, it was likely that viable organisms or granulomas would be identified; this would also provide no further information regarding whether he had indeed developed systemic BCG‐osis. In light of his presentation, investigations, and clinical progression, a clinical diagnosis of systemic BCG‐osis was made following discussion with the Infectious Diseases team.

As a result, broad‐spectrum antibiotic therapy was stopped and he was started on rifampicin, isoniazid and ethambutol, as well as ciprofloxacin for Gram‐negative mycobacterial cover, and oral steroids. The patient felt markedly better following treatment with this regimen. His case was discussed with Haematologists, who felt that he may benefit from a bone marrow trephine to exclude bone marrow involvement. The bone marrow biopsy revealed reactive features and several small granulomas.

The patient continued to improve and was deemed medically fit for discharge on his remaining course of antituberculous treatment with a long‐term plan to undergo a radical cystectomy once sufficiently recovered from this episode of systemic BCG‐osis.

## Discussion

Bacillus Calmette‐Guérin immunotherapy has a crucial role in the treatment of NMIBC following TURBT; maintenance BCG has been demonstrated to be superior to TURBT alone or intravesical mitomycin C at reducing the risk of either disease recurrence or progression 6, 9. However, intravesical BCG treatment does not come without its side effects and complications. Due to the inflammatory response within the bladder, dysuria, and frequency (5–90% incidence), hematuria (1–34% incidence) and fever (3% incidence) are not uncommon and often occur within 48 h postinstillation 10.

Systemic complications occur less frequently, but are usually much more severe. These include granulomatous prostatitis, granulomatous epididymo‐orchitis, arthritis, and migratory arthralgia. Disseminated BCG infection may also involve granulomatous hepatitis, pneumonitis, and involvement of bone marrow, and could lead to severe sepsis progressing to multisystem organ failure 11, 12. Recent estimates suggest that one in 15,000 patients receiving intravesical BCG develops a septic reaction (0.0067% incidence) 12.

Risk factors increasing the risk of systemic side effects include bladder trauma, cystitis, immunosuppression, diabetes, and genetics 10. In the case of our patient, his most recent instillation prior to onset of symptoms was noted to involve a traumatic catheterization with bleeding; this may have resulted in the initial spread of BCG at this time. Bladder trauma appears to allow intravesical mycobacteria to access the systemic circulation, resulting in systemic BCG‐osis 13.

The exact cause of systemic BCG‐osis is still under investigation. Some theories suggest that this may simply be due to the systemic spread and dissemination of *Mycobacterium* spp., with some authors identifying viable organisms in tissue 14. It has also been postulated that this condition is due to a systemic type IV hypersensitivity reaction to BCG; this is based upon findings of granulomas on histology, negative blood and tissue cultures, as well as negative Ziehl‐Neelsen staining 15, 16. With our patient, his case appears to support the theory that systemic BCG‐osis is due to a hypersensitivity reaction following systemic dissemination. This is demonstrated by negative cultures and stains, hypergranular neutrophils suggesting reactive cells on blood films, and a dramatic response to steroids.

There are currently no standard guidelines for management of disseminated BCG‐osis 17. A literature review by Huang recommended that patients on BCG immunotherapy who develop a high‐grade fever (defined as temperature above 39°C) should be admitted to hospital for monitoring of signs of BCG sepsis 18. In the meantime, BCG immunotherapy should be discontinued and antituberculosis therapy, in conjunction with steroid therapy, should be commenced as empirical treatment. Antituberculous therapy should be continued for 3–6 months, while steroid treatment should be gradually tapered after patients demonstrate a clinical response. This is vital as abrupt termination of steroid therapy has been observed to result in exacerbation of systemic BCG‐osis. The decision to continue BCG immunotherapy should then be made based on individual risk‐benefit evaluation 12, 18.

In summary, this case demonstrates the importance of being aware of severe complications that are associated with BCG immunotherapy in bladder cancer and highlights the value of a multidisciplinary approach to best optimize patient care.

## Authorship

FL: involved in conception of the work, data collection, data analysis and interpretation, preparation of manuscript, literature review, and revision of manuscript. YT: made preparation of manuscript, literature review, and revision of manuscript. DH: involved in data interpretation and supervision.

## Conflicts of Interest

None declared.
